# Persistent neurocognitive abnormalities as clinical sequelae of mild to moderate COVID-19

**DOI:** 10.3389/fmed.2025.1429529

**Published:** 2025-09-03

**Authors:** Agnieszka Bednarska, Marek Radkowski, Tomasz Laskus, Dominik Bursa, Natalia Bluszcz, Michał Makowiecki, Iwona Sosińska-Bryła, Marcin Paciorek, Dawid Porowski, Małgorzata Hackiewicz, Justyna Kowalska, Anna Furman-Dłubała, Andrzej Horban

**Affiliations:** ^1^Department of Adults' Infectious Diseases, Medical University of Warsaw, Warsaw, Poland; ^2^Hospital for Infectious Diseases in Warsaw, Warsaw, Poland; ^3^Department of Immunopathology of Infectious and Parasitic Diseases, Medical University of Warsaw, Warsaw, Poland; ^4^Center for Observational Research in Infectious Diseases, Medical University of Warsaw, Żwirki i Wigury, Warsaw, Poland

**Keywords:** mild to moderate, COVID-19, persistent symptom, neurocognitive, anti-SARS-CoV-2 antibodies

## Abstract

**Objectives:**

This study aimed to examine the incidence of symptoms that indicate neurological or pulmonary complications after recovery from mild to moderate COVID-19.

**Methods:**

The study included 138 adult outpatients who underwent testing that included chest X-ray (CXR), complete blood count (CBC), C-reactive protein (CRP), interleukin 6 (IL-6), and D-dimer assessments during the acute phase of the illness. In addition, 30 days after being classified as convalescent, serological tests for IgM and IgG antibodies against severe acute respiratory syndrome coronavirus 2 (SARS-CoV-2) were performed, and the patients were asked to complete a survey assessing their overall wellbeing.

**Results:**

The most common sequelae included decreased physical efficiency (35%), weakness (24%), difficulty concentrating (16%), and memory problems (15%), which were correlated with abnormal chest X-ray findings. Seroconversion to anti-SARS-CoV-2 IgG was detected in 49 (87.5%) out of 56 patients tested and was more common among those with a more severe course of the infection.

**Conclusion:**

Individuals with mild to moderate COVID-19 are likely to experience persistent neurocognitive symptoms. Patients with initial abnormal chest X-ray findings and elevated inflammatory parameters are more likely to seroconvert to anti-SARS-CoV-2.

## Introduction

In late 2019, a novel and highly transmissible coronavirus known as severe acute respiratory syndrome coronavirus 2 (SARS-CoV-2) emerged in the city of Wuhan in China (1). Patients with SARS-CoV-2 infection can experience a wide range of clinical manifestations, which can vary from being asymptomatic to experiencing critical illness (2). Although most individuals with mild to moderate illness can be managed at home, they may exhibit significant variability in terms of inflammatory reactions, pulmonary manifestations, and the expression of protective antibodies (3, 4).

Persistent complications of COVID-19 can affect the function of several organs, including the lungs, heart, and brain. Commonly reported symptoms such as persistent cough, dyspnea, and fatigue (5–7) can last for weeks, months, or even years (8). More than half of COVID-19 survivors report experiencing at least one lingering effect, known as a sequela, 1 month after infection. The most prevalent sequelae include pulmonary complications and neurological or mental health disorders, such as functional mobility impairments, difficulty concentrating, generalized anxiety disorder, overall functional impairment, and fatigue or muscle weakness (9).

While numerous studies have investigated the outcomes of severe COVID-19 infections, research on the recovery of patients from less severe cases remains limited. In this study, we followed up outpatients with a mild to moderate course of COVID-19 and examined the incidence of symptoms indicating neurological or pulmonary complications following their recovery from the acute infection. The impact of initial chest X-ray (CXR) findings, laboratory parameters, and seroconversion to anti-SARS-CoV-2 IgG was evaluated.

## Methods

We followed up adult patients (≥ 18 years old) who were diagnosed with COVID-19 between 1 June 2020 and 6 July 2020 at the Emergency Department of the Infectious Disease Hospital in Warsaw, Poland.

Patients with suggestive symptoms were tested for COVID-19 using the reverse transcription polymerase chain reaction (RT-PCR). Individuals with a positive RT-PCR test for SARS-CoV-2 from a nasopharyngeal swab and who did not require hospitalization (no oxygen therapy needed) were enrolled in the study and invited for an assessment visit within 3–7 days. Testing for the presence of SARS-CoV-2 RNA in nasopharyngeal swabs was performed every 7 days. Patients with two sequential negative RT-PCR results were classified as convalescent and scheduled for an appointment 30 days later. On average, it took 69 days (38–107, median 69) from the initial diagnosis to recovery.

During the initial visit, the patients underwent a chest X-ray (CXR) and the following blood tests: complete blood count (CBC), C-reactive protein (CRP), interleukin 6 (IL-6), and D-dimer. The chest X-ray included posterior–anterior (PA) and lateral projections, which were independently analyzed by two radiology specialists.

Although chest X-ray are characterized by lower sensitivity compared to chest computed tomography (CT), it was selected due to their accessibility and speed, especially for ambulatory patients (10). This strategy was in line with the recommendations of the American College of Radiology and the European Society of Radiology for COVID-19 screening and diagnosis at that time (11).

During the last visit serological tests (IgM and IgG anti SARS-CoV-2) were performed and patients were asked to complete a survey assessing their wellbeing which included: general weakness, decreased physical efficiency, dyspnea while resting, dyspnea during physical activity, cough, loss of smell, loss of taste, burning of the skin, itchy skin, arthralgia, problems with concentration and memory (Supplementary Figure 1).

### Statistical analysis

The distribution of variables was analyzed using the Shapiro–Wilk test. In the absence of a normal distribution of variables, the results were presented as median values and interquartile ranges (Md and IQR). The Mann–Whitney U test and the chi-squared test were performed to compare the groups. Variables associated with an increased likelihood of pneumonia and the occurrence of certain symptoms were identified using multivariate logistic regression analysis. The construction of the multivariate model was based only on those variables whose *p*-value of significance in the univariate analysis was not greater than 0.1. The level of statistical significance was set at a *p*-value of <0.05. All analyses were performed using Statistica software, version 13.1 (StatSoft), with a medical add-on.

## Results

Of the 3,185 patients evaluated at the Emergency Department of the Infectious Disease Hospital in Warsaw, Poland, 554 tested positive for SARS-CoV-2. Of these, 138 patients (65 women and 73 men) who did not require hospitalization were included in the study. The median age at diagnosis was 40 years (age range: 18–82 years). The majority of individuals were recruited in June 2020, with only one patient enrolled in July 2020. They were followed up until 25 September 2020. On average, 69 days (38–107, median 69) passed from initial diagnosis to recovery (Table 1).

**Table 1:** Baseline characteristics of the study population.

Demographics	Study population *n* = 138
Age, years, mean (SD)	46 (16.16)
Women, gender *n* (%)	65 (47%)
Men, gender *n* (%)	73 (53%)
Abnormalities in the initial chest X-ray, *n* (%)	42 (30%)
Abnormal parameter results (standard range)	Study population *n* = 138, mean (SD)
CRP (< 10 mg/L)	27.1 mg/L (41.9)
IL-6 (< 7 pg./mL)	12.1 pg./mL (20.3)
D-dimer (< 500 ng/mL)	622.6 ng/mL (711.8)
Hgb (13.7–16.7 g/dL)	14.2 g/dL (1.6)
PLT (125–396 G/L)	247.3 G/L (105.9)
WBC (3.98–10.04 10^3^/ul)	6.0 10^3^/ul (1.8)
Characteristics	Study population *n* = 56
Seroconversion to anti-SARS-CoV-2 IgG, *n* (%)	49 (88%)
Duration from initial diagnosis to recovery, days, mean (SD)	69 (16.5)
Symptoms lasting more than 12 weeks (out of 45 patients), *n* (%)	13 (29%)
Symptoms	Study population *n* = 55
Decreased physical efficiency, *n* (%)	19 (35%)
Weakness, *n* (%)	13 (24%)
Difficulty concentrating, *n* (%)	9 (16%)
Memory problems, *n* (%)	8 (15%)
Dyspnea during physical activity, *n* (%)	6 (11%)
Persistent cough, *n* (%)	6 (11%)
Loss of smell, *n* (%)	5 (9%)
Arthralgia, *n* (%)	5 (9%)
Loss of taste, *n* (%)	3 (5%)
Itchy skin, *n* (%)	2 (4%)

CRP, C-reactive protein; IL-6, interleukin 6; Hgb, hemoglobin; PLT, platelet count; WBC, white blood cell count.

In 42 (30%) patients, the initial chest X-rays revealed abnormalities indicative of COVID-19 pneumonia. These patients were statistically older (*p* < 0.001), had higher concentrations of CRP (*p* < 0.001), IL-6 (*p* < 0.001), and D-dimer (*p* < 0.001), and exhibited lower concentrations of hemoglobin (*p* = 0.006) compared to those without pneumonia (Table 2).

**Table 2:** Age and laboratory test results in patients with normal or pneumonia-related changes on the initial chest X-ray.

Parameter	Normal		Pneumonia		*p*-value
	Median	IQR	Median	IQR	
Age	38.00	20.00	56.00	22.00	0.000029
CRP (mg/l)	6.00	8.00	28.00	46.00	0.000005
IL-6 (pg/ml)	1.50	1.30	10.75	29.70	0.000006
D-dimer (ng/ml)	275.32	195.54	692.87	756.73	<0.000001
Hemoglobin (g/dl)	15.00	2.30	13.95	2.30	0.006
Platelets (G/l)	229.00	88.00	254.50	134.00	0.21
Leukocytes (G/l)	5.60	2.20	6.35	2.60	0.07

Analysis performed using the Mann–Whitney U test; CRP, C-reactive protein; IL-6, interleukin 6.

**Figure 1:**
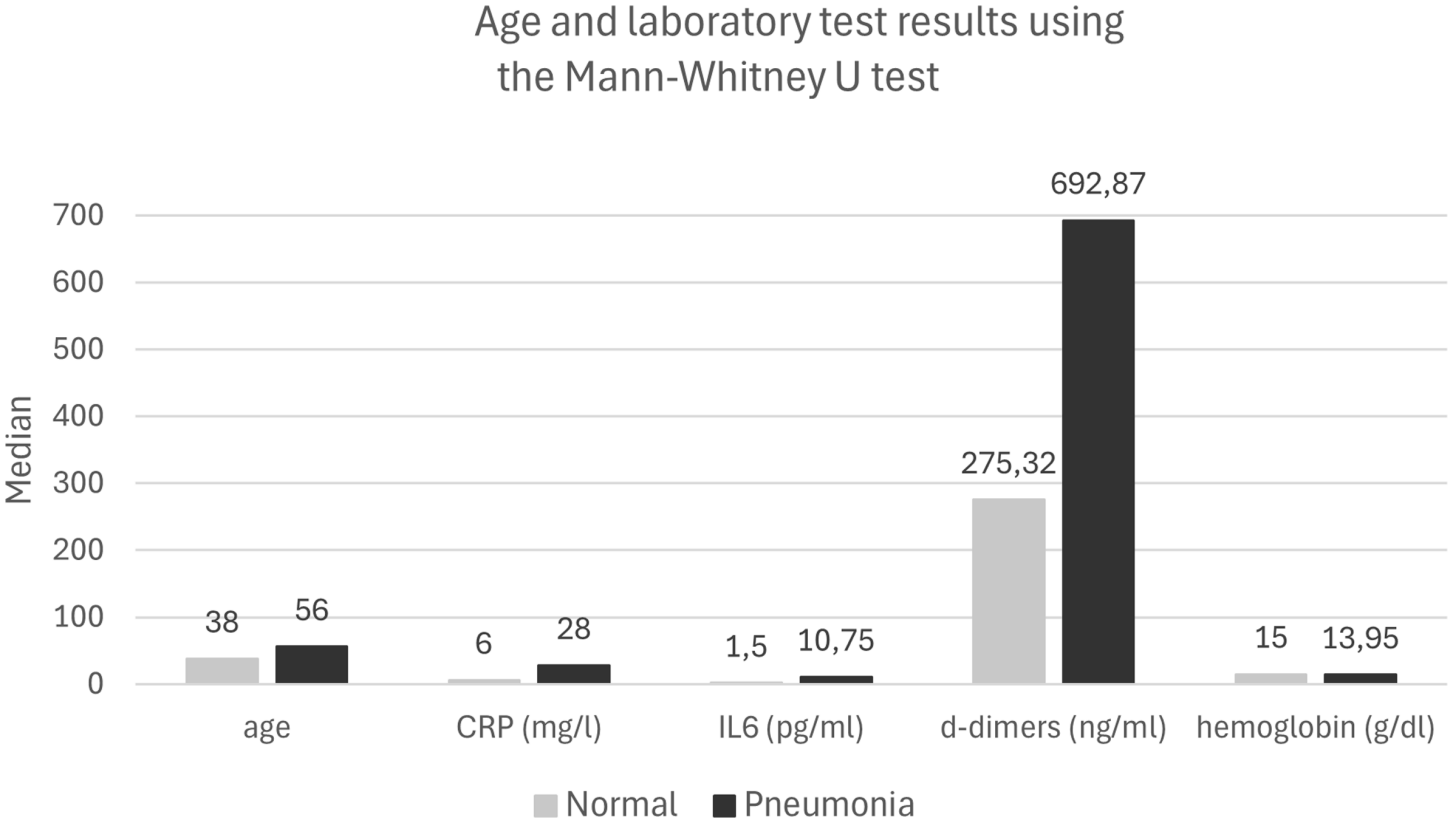
Age and laboratory test results using the Mann-Whitney U test.

The univariate logistic regression analysis revealed that age (over 60 years), CRP, IL-6, D-dimer, hemoglobin concentrations, and platelet count were associated with an increased risk of pneumonia. However, in the multivariate logistic regression model, only CRP concentration was identified as an independent risk factor for pneumonia (OR = 1.062; 95% Cl 1.008–1.119; *p* = 0.023) (Table 3; Figure 1).

**Table 3:** Association between variables and abnormal chest X-ray results in the univariate logistic regression analysis.

Variables	OR	95% CI	*p*-value
Gender	0.792	0.361–1.738	0.5604
Age	1.057	1.028–1.087	0.0001
18–29 years	1.000	-	-
30–39 years	1.875	0.298–11.780	0.503
40–49 years	6.429	1.217–33.971	0.0285
50–59 years	5.625	0.915–34.574	0.0623
60–69 years	18.000	2.955–109.662	0.0017
≥ 70 years	17.500	2.365–129.510	0.0051
CRP (mg/l)	1.063	1.030–1.098	0.0002
IL-6 (pg/ml)	1.076	1.027–1.128	0.0021
D-dimer (ng/ml)	1.003	1.001–1.005	0.0002
Hemoglobin (g/dl)	0.663	0.494–0.888	0.0059
Platelets (G/l)	1.004	1.000–1.009	0.0458
Leukocytes (G/l)	1.260	0.990–1.603	0.0602

CRP, C-reactive protein; IL-6, interleukin 6; OR, odds ratio; CI, confidence interval.

In the survey completed by the convalescents (*n* = 55), decreased physical efficiency (*n* = 19, 35%), weakness (*n* = 13, 24%), difficulty concentrating (*n* = 9, 16%), and memory problems (*n* = 8, 15%) were the most commonly reported symptoms. These symptoms were followed by dyspnea during physical activity (*n* = 6, 11%), persistent cough (*n* = 6, 11%), loss of smell (*n* = 5, 9%), arthralgia (*n* = 5, 9%), loss of taste (*n* = 3, 5%), and itchy skin (*n* = 2, 4%). A total of 33 patients had no complaints about their health, and 5 patients reported only one symptom (3 of them reported decreased physical efficiency). Dyspnea during physical activity was the only symptom that was statistically associated with older age (*p* = 0.03).

**Figure 2:**
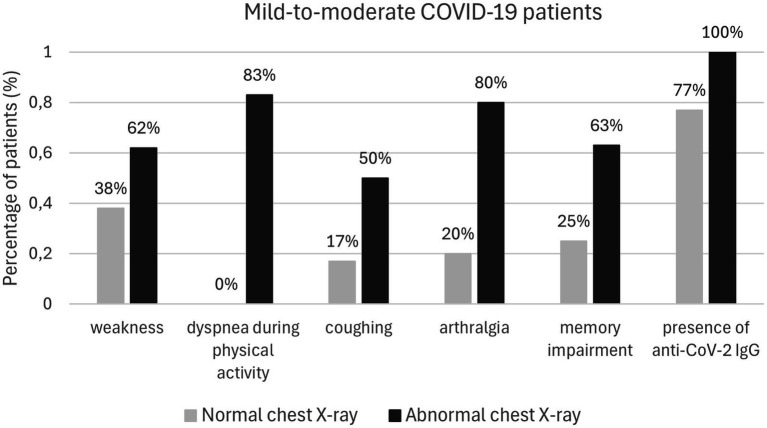
Mild-to-moderate COVID-19 patients.

Patients with initial abnormal chest X-ray findings were more likely to experience weakness (*p* = 0.04), dyspnea during physical activity (*p* = 0.008), arthralgia (*p* = 0.008), memory problems (*p* = 0.017), and persistent cough (*p* = 0.019). Furthermore, women were more likely to report persistent cough (*p* = 0.048) and difficulty concentrating (*p* = 0.033; Table 4; Figure 2).

**Table 4:** Symptoms reported by convalescents (*n* = 55) 30 days after the virus elimination based on chest X-ray results and gender.

Symptoms	Patients with or without pneumonia			Gender		
	Normal chest X-ray *n* = 61	Abnormal chest X-ray *n* = 42	*p*-value	Woman *n* = 65	Man *n* = 73	*p*-value
Weakness (*n* = 13)	5 (38%)	8 (62%)	0.040	7	6	0.381
Decreased physical efficiency (*n* = 19)	8 (42%)	10 (53%)	0.066	11	8	0.178
Dyspnea while resting	-	-	-	-	-	-
Dyspnea during physical activity (*n* = 6)	0	5 (83%)	0.008	4	2	0.269
Persistent cough (*n* = 6)	1 (17%)	3 (50%)	0.019	5	1	0.048
Loss of smell (*n* = 5)	3 (60%)	2 (40%)	0.832	2	3	0.797
Loss of taste (*n* = 3)	2 (67%)	1 (33%)	0.689	2	1	0.448
Burning of the skin	-	-	-	-	-	-
Itchy skin (*n* = 2)	0	1 (50%)	0.258	2	0	0.115
Arthralgia (*n* = 5)	1 (20%)	4 (80%)	0.008	4	1	0.104
Difficulty concentrating (*n* = 9)	3 (33%)	5 (56%)	0.055	7	2	0.033
Memory problems (*n* = 8)	2 (25%)	5 (63%)	0.017	6	8	0.069

Analysis was performed using the chi-squared test; % = percentage of individuals reporting a symptom.

A total of 49 (87.5%) out of 56 tested patients revealed the presence of anti-SARS-CoV-2 IgG antibodies in the blood.

**Figure 3:**
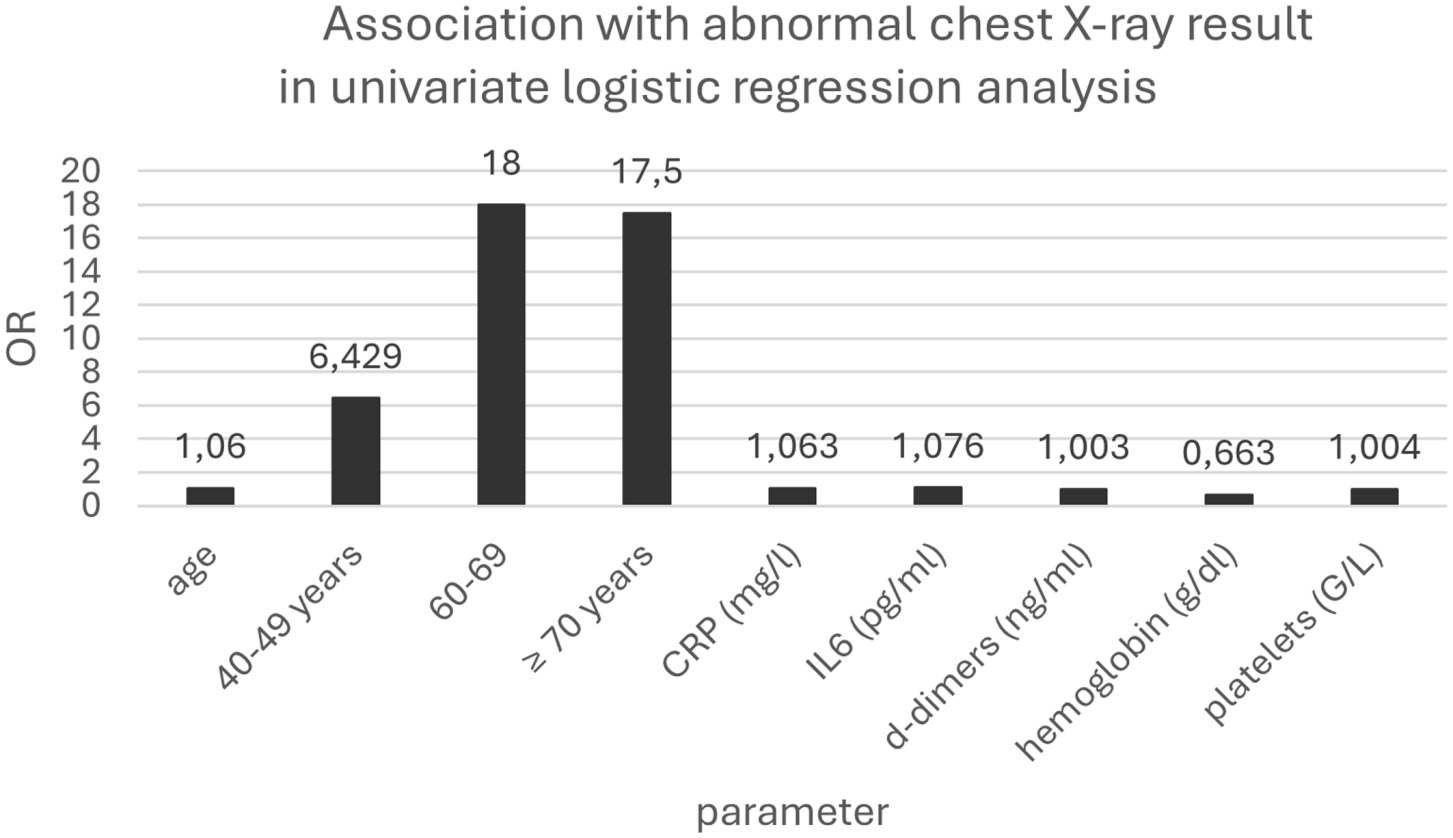
Association with abnormal chest X-ray result in univariate logistic regression analysis.

The patients with pneumonia, compared to those with normal chest X-ray results, were more likely to be positive for anti-SARS-CoV-2 IgG (100% vs. 77%, *p* = 0.021). Furthermore, seroconversion was more common in the patients with elevated levels of CRP (*p* = 0.023), IL-6 (*p* = 0.004), and D-dimer (*p* = 0.0005), as well as in the older patients (*p* = 0.006). There was no difference in anti-SARS-CoV-2 seroconversion between genders (*p* = 0.31118; Table 5; Figure 3).

**Table 5:** Characteristics of patients with or without seroconversion to anti-SARS-CoV-2 IgG.

Parameter	Anti-SARS-CoV-2 IgG				*p*-value
	Positive result		Negative result		
	Median	IQR	Median	IQR	
CRP (mg/l)	17.00	34.00	6.00	1.00	0.02
IL-6 (pg/ml)	5.80	18.15	1.50	0.00	0.004
D-dimer (ng/ml)	459.98	544.34	182.42	69.57	0.0005
Hemoglobin (g/dl)	14.25	2.30	15.35	2.20	0.20
Platelets (G/l)	234.00	123.00	221.00	19.00	0.77
Leukocytes (G/l)	5.75	2.30	4.70	3.00	0.54
Age (years)	47.00	23.00	31.00	16.00	0.006

Analysis was performed using the Mann–Whitney U test; CRP, C-reactive protein; IL-6, interleukin 6; IQR, interquartile range.

Neither laboratory parameters, age, nor chest X-ray results appeared to be a predisposing factor for seroconversion in the univariate and multivariate analyses.

## Discussion

Post-acute sequelae can affect convalescents regardless of their age or the severity of infection, including non-hospitalized patients (12). Earlier findings suggest that up to 56% of patients hospitalized for COVID-19 experience neuropsychiatric complications 1 month after discharge, and the incidence of psychiatric illness within 14 and 90 days after SARS-CoV-2 infection is estimated at 18% (13, 14). Persistent hypoxemia was reported in approximately 6% of patients, chest pain was experienced in 20%, and ongoing myocardial inflammation was present in up to 60% of post-acute COVID-19 patients at the two-month follow-up (15).

The main aim of our study was to correlate symptoms and routine test results with pulmonary and neurological sequelae in 138 patients diagnosed with mild or moderate SARS-CoV-2 infection. In addition, we analyzed the prevalence of pneumonia during the acute phase of infection and seroconversion to anti-SARS-CoV-2 30 days after viral clearance.

Previous studies have reported general symptoms such as weakness and fatigue as the most common symptoms among post-COVID-19 patients, followed by mental, cardiopulmonary, and neurological symptoms (16, 17). After severe forms of COVID-19, post-infection complications are common and pronounced; for example, cognitive impairment has been reported to be more prominent in patients with more severe forms of disease (18) and prolonged hospital stays (17). However, individuals with moderate or mild disease who stayed at home during the acute phase and did not require oxygen support were not free from sequelae (19).

In our study, the most common sequelae of COVID-19 were decreased physical efficiency (35%), weakness (24%), difficulty concentrating (16%), and memory problems (15%), all of which correlated with abnormal initial chest X-ray results. The chest X-ray results revealed changes indicative of COVID-19 pneumonia in 42 (30%) patients. Patients with pneumonia were statistically older and had higher levels of inflammatory markers (CRP, IL-6, and D-dimer).

Persistent inflammation caused by viral persistence, immune dysregulation, or autoimmunity can be the cause of continuing symptoms following COVID-19 (20, 21), and mild or moderate infection can also trigger a prolonged immune response (22). Furthermore, it has been observed that the development of pneumonia delays viral clearance, which could, in turn, cause residual immunological alterations (23, 24).

Neurological and cognitive dysfunction after COVID-19 may result from direct damage to the central nervous system (25). The hippocampus, which serves as a critical memory center (26), is particularly vulnerable to SARS-CoV-2 infection (25). The virus has been found to infect brain cells, stimulate glial cells, and induce a pro-inflammatory state in the brain (7, 27). SARS-CoV-2 binds to the cells via the S1 subunit of its spike protein, and it has been shown that radioiodinated S1, when administered intravenously and intranasally, has the ability to cross the blood–brain barrier and enter the brain (28). Neurocognitive complications are seen in both severe and mild COVID-19 cases. Neocortical brain degeneration—primarily affecting the fronto-parietal brain and the right thalamus—has been detected in individuals who recovered from SARS-CoV-2 infection. Although this degeneration was more pronounced in those with severe infection, it was also present in patients with asymptomatic or mild COVID-19 (29).

As mentioned above, we also analyzed seroconversion to anti-SARS-CoV-2 IgG antibodies 30 days after viral clearance. Anti-SARS-CoV-2 IgG antibodies were detected in 49 out of 56 tested patients (87.5%), and this seroconversion was statistically more common in patients with a more severe course of COVID-19 (as indicated by abnormal chest X-ray results and higher CRP and D-dimer concentrations).

There is a positive correlation between the severity of the disease and the prevalence and titers of anti-SARS-CoV-2 IgG antibodies (30, 31). Huan Ma et al. showed that antibody levels in patients with moderate to severe COVID-19 were higher compared to those with mild disease (32). The prolonged course of the disease, along with longer exposure to the virus, may also provide a timeframe for enhanced antibody affinity maturation (33).

In the study by Young HS et al., which involved individuals with asymptomatic disease, younger participants had a lower rate of antibody positivity than older participants (34). However, the opposite was observed in our cohort, which could be the result of a more severe course of infection among older adults (35).

Our study is not without limitations. Of these, the lack of information about comorbidities and the low number of returned questionnaires appear to be the most significant.

In summary, we found that elevated basic inflammatory parameters, particularly C-reactive protein concentration, are associated with the development of pneumonia and that patients with abnormal chest X-ray findings and elevated inflammatory parameters are more likely to seroconvert to anti-SARS-CoV-2 antibodies. Furthermore, outpatients with mild to moderate COVID-19 are likely to develop persistent neurological and neurocognitive symptoms.

## Data Availability

The data analyzed in this study is subject to the following licenses/restrictions: contain information that can identify indivudals. They are stored in protected files and requests to access these datasets should be directed to Agnieszka Bednarska abednarska@zakazny.pl.
