# Supplementary material for: Persistent neurocognitive abnormalities as clinical sequelae of mild to moderate COVID-19
Source: Front Med (Lausanne). 2025 Sep 3;12:1429529. doi: 10.3389/fmed.2025.1429529 (PMC12440792; doi:10.3389/fmed.2025.1429529)
Supplement: Supplementary file 1 [file Supplementary_file_1.docx]

| **Did you have any of the symptoms after being acknowledge as convalescent:** | **YES** | **IF YES – FOR HOW LONG**  **(numbers of days or weeks; if still present please write still present)** | **NO** |
| --- | --- | --- | --- |
| WEAKNESS |  |  |  |
| DECREASED PHYSICAL EFFICIENCY |  |  |  |
| DYSPNEA WHILE RESTING |  |  |  |
| DYSPNEA DURIGN PHYSICAL ACTIVITY |  |  |  |
| COUGHING |  |  |  |
| LOSS OF SMELL |  |  |  |
| LOSS OF TASTE |  |  |  |
| BURNING OF THE SKIN |  |  |  |
| ITCHY SKIN |  |  |  |
| ARTHRALGIA |  |  |  |
| DIFFICULTY CONCENTRATION |  |  |  |
| DIFFICULTY REMEMBERING |  |  |  |
